# Introduction of virtual microscopy in routine surgical pathology - a hypothesis and personal view from Europe

**DOI:** 10.1186/1746-1596-7-48

**Published:** 2012-04-30

**Authors:** Klaus Kayser

**Affiliations:** 1 Director, UICC-TPCC, Institute of Pathology, Charite, Charite Platz 1, D-10117, Berlin, Germany

## Abstract

The technology of whole image acquisition from histological glass slides (Virtual slides, (VS)) and its associated software such as image storage, viewers, and virtual microscopy (VM), has matured in the recent years. There is an ongoing discussion whether to introduce VM into routine diagnostic surgical pathology (tissue-based diagnosis) or not, and if these are to be introduced how best to do this. The discussion also centres around how to substantially define the mandatory standards and working conditions related to introducing VM. This article briefly describes some hypotheses alongside our perspective and that of several of our European colleagues who have experienced VS and VM either in research or routine praxis. After consideration of the different opinions and published data the following statements can be derived: 1. Experiences from static and remote telepathology as well as from daily routine diagnoses, confirm that VM is a diagnostic tool that can be handled with the same diagnostic accuracy as conventional microscopy; at least no statistically significant differences (*p* > 0.05) exist. 2. VM possesses several practical advantages in comparison to conventional microscopy; such as digital image storage and retrieval and contemporary display of multiple images (acquired from different stains, and/or different cases). 3. VM enables fast and efficient feedback between the pathologist and the laboratory in terms of ordered additional stains, automated access to the latest research for references, and fast consultation with outstanding telepathology experts. 4. Industry has already invested “big money” into this technology which certainly will be of influence in its future development. The main constraints against VM include the questionable reimbursement of the initial investment, the missing direct and short term financial benefit, and the loss of potential biological identity between the patient and the examined tissue. This article tries to analyze and evaluate the factors that influence the implementation of VM into routine tissue-based diagnosis, for example in combination with predictive diagnosis. It focuses on describing the advantages of modern and innovative electronically based communication technology.

**Virtual Slides:**

The virtual slide(s) for this article can be found here: http://www.diagnosticpathology.diagnomx.eu/vs/1245603103708547

## Introduction

Tissue based diagnosis derived from human tissue(s) still remains the most reliable method of disease recognition and classification in both sensitivity and specificity [1]. For example, in the USA only 272 surgical pathology claims from 1998–2003 have been reported to have significant therapeutic consequences and an inter-observer error rate of 80–95% dependent upon technical procedure (fine needle aspiration, etc.), and diagnosis (Spitz nevus versus melanoma, etc.) [2,3]. All these studies rely on manual workflow and manual tissue processing. Considerations on the impact of digitised tissues or slides, and electronic communication in terms of expert consultation, have not been included up until now; to our knowledge.

The new technology of image acquisition and digitisation (virtual slides, (VS)) has been broadly applied in nearly all Institutes of Pathology related to Universities in Germany and other European countries. It has been used for education, storage of rare cases, clinical pathological conferences, and research assessment such as tissue micro arrays (TMA) [4,5]. It’s implementation into the routine research workflow is still in its initial stages. Some private institutes of pathology are already reporting satisfying and good results when deriving diagnoses from routinely acquired VS [6-8].

Which standards and control functions need to be fulfilled by the corresponding laboratories and technical equipment specialists in order to ascertain or corroborate the high level of quality of diagnostic surgical pathology in VM?

### Conventional laboratory workflow and tissue-based diagnosis

Human tissue needs to be fixed, processed, cut, stained, and finally analyzed by a surgical pathologist. Specific equipment is commonly used for fixation, tissue processing, and staining, which is loaded manually [9,10]. Microtome cuts usually require manual performance. The whole workflow is monitored by a laboratory information system (LIS), which itself is subject to accreditation and certification [11]. Capable LIS report the stages of individual tissues at any time and step of the workflow [12,13]. The quality of the obtained slides varies. Slides of good quality allow easier and more precise diagnoses; although experienced pathologists well versed in specific laboratory parameters can view slides of poorer quality with similar diagnostic accuracy. This statement, however, only holds true for “common H&E based” diagnoses, such as tuberculosis or squamous cell carcinoma.

The demands of guiding an adequate therapy have led to the term “tissue–based diagnosis” which includes H&E based diagnosis, prognosis associated diagnosis (usually demanding immunohistochemical investigations (IHC)), predictive diagnosis (usually demanding IHC and gene mutation analysis (PCR)), and risk estimation, which is the estimation of risk prior to the establishment of a certain disease (usually demanding gene analysis (PCR)) [14,15]. Prognosis associated diagnosis, predictive diagnosis, and risk estimation are more sensitive to variations in technical performance in comparison to H&E based diagnosis. They have to be continuously monitored and are sensitive to the fixation medium [16]. Derived from the ring investigations in clinical pathology, IHC standard stained slides are distributed and judged by referees in several European countries (Germany, Great Britain, and others) [17,18]. The diagnostic accuracy is controlled by distributing VS to surgical pathologists and evaluating their responses via electronic communication [13,19].

When analyzing the laboratory workflow, a principal component has to be included: the primary H&E diagnosis has to be confirmed and further refined by additional laboratory procedures such as additional stains like PAS or Giemsa, etc., IHC or additional investigations such as PCR, or in situ hybridization, etc. In a laboratory with a conventional workflow they are ordered by the responsible pathologist and depend upon the demands of the clinician. All these actions can be aggregated under the topic reliable “communication” and user – friendly “man – machine interaction”. Herein, appropriate digital compartments are essential.

### Digital pathology and tissue-based diagnosis

The acquisition of virtual slides and associated virtual microscopy (VM) to interpret digital images occur close to the end of the diagnostic chain, typically just before the diagnosis is reported. It is useful to distinguish between interactive and automated VM [20]. Interactive VM is the pathologist’s diagnostic performance when viewing VS. Automated VM includes the implementation of computer-aided “diagnostic assistants” [21]. They can be compared with program assistants that assist in detecting spelling errors in a letter, or in formatting certain documents, etc. [21,22]. Most vendors concentrate on interactive VM. Some add IHC measurement programs that have been designed to calculate IHC scores such as Her2/neu or hormone receptor status in breast cancer [23]. Outside of gynaecological cytopathology, automated VM has not been fully implemented to the best of my knowledge.

Obviously, the quality of VS depends upon the original slides and upon the colour and spatial accuracy of the whole slide imaging (WSI) scanner [13,23] which is used. Most WSI scanners can be equipped with objectives of different magnification (i.e., x10, x20, and x40) which define the scanning time and mainly the number of focal z-planes. In histology (cellular structure based morphology) one individual plane is usually sufficient to render a diagnostic interpretation, in contrast to cytology (intra-cellular structure based morphology) which requires several focal planes (referring to the Z-axis) [13].

Implementing VM into routine practice offers several principle advantages, which can be summarized as follows:

 1. Once a scanner delivers VS of sufficient diagnostic quality, the technology generally continues to produce images of similar quality. Working with a conventional microscope requires frequent “resetting” of the focus plane, adjustment of brightness, etc. Conventional microscope settings also depend upon personal preferences, which can be simulated by VS viewers if desired.

 2. Several images obtained from different stains of the same case or tumor can be viewed simultaneously with VM. This feature allows for a more precise judgement of prognosis associated diagnosis and predictive diagnosis.

 3. Quantitative evaluation of object and structure features can be evaluated either interactively or automatically, which is of assistance in prognosis associated diagnosis and predictive diagnosis.

 4. Annotations can be included for teaching, marking areas of interest, or discussion purposes in VM.

The advantages of maintaining the conventional performance of tissue-based diagnosis, which are identical with the constraints of the implementation of VM include:

 1. The lack of financial advantage of VM implementation in conjunction with the decreasing prices for hardware and software. This is in direct contrast to the financial advantage of implementing digital imaging on live images in disciplines such as radiology, which saves the expenses of paper based diagnosis (films). The investigations are not negligible and can amount to about 20% of the gross budget dependent upon the size of the pathology institute (private estimation). There seem to be no savings when implementing VM, because the processes in the laboratory mainly remain untouched and only the workflow is affected. Some authors however have mentioned the comparable save in labour when retrieving a case and the increased safety of diagnoses using VM [6,8]. However, these are only secondary savings, and mainly depend upon the local environment [4,24].

 2. Security of the patient’s diagnosis, and the possibility to control the origin of the glass slides by finger printing the patient’s DNA at any time. Obviously, this cannot be stated in VS, however, modern electronic safety controls permit an unambiguous relationship between the VM and the original glass slides [25].

 3. Additional arguments such as difficulties to view and diagnose VS compared to conventional microscopes depend more or less on training and performance of the individual pathologist [24,26,27]. This has also been demonstrated in teaching courses using VS and conventional microscopes for medical students in anatomy [24,28-31]. If appropriately trained, most of the students in these courses preferred VS compared to receiving training with conventional microscopes [12].

In summary, VM offers greater advantages than conventional light microscopy with respect to prognosis associated diagnosis and predictive diagnosis, i.e., for those types of diagnoses which are closely associated with guiding patient therapy.

### Consecutives

Tissue-based diagnosis still remains related to the pathologist’s knowledge in both conventional microscopy and VM. The diagnosis information sources include the patient’s history, microscopic and gross images, and additional findings (live imaging, etc.). Microscopic images including VS contribute significantly, however not in total, to the patient’s diagnosis. The more distinct a diagnosis, the more information in addition to microscopic images is required for an adequate diagnosis to be made [13,32-34].

The optical pathways are important to obtain good images, but are not the only prerequisite. Preservation, fixation, and embedding of tissue, cutting of tissue blocks, and staining of glass slides also contribute to VS quality to a high degree. From our experience in Europe, the WSI scanner performance (i.e. the acquisition of VS) is of the highest quality to permit diagnostic interpretations to be made.

What can we conclude?

 1. The histological diagnosis is based not only on the final digital image, but also upon a chain of laboratory procedures with additive external clinical information.

 2. Diagnoses have to be delivered at different levels in relation to the clinical demands and potential treatment needs of the patient.

 3. “Crude diagnoses”, for example H&E diagnoses, are adequate in many situations, for example in odontogenic cysts, acute appendicitis, common nevi, etc.

 4. Adequate quality control of histological diagnoses should start with the final outcome, i.e., analysing the diagnostic quality of the material being viewed in relation to the demands of potential therapy.

 5. The weakest link in the diagnostic chain should be addressed with high priority. It can vary - for example, tissue preservation and fixation are subject to a broad variance.

 6. The optical pathway is the end link in the diagnostic workflow process, and therefore, of less significance to associated laboratory reprocessing such as IHC, ISH, etc.

 7. Monitoring intra-laboratory procedures (e.g., efficiency and reproducibility of reprocessing and staining, etc.) should be separated from the evaluation of diagnosis quality.

 8. Evaluation of diagnostic errors should take into account the therapeutic consequences and the local environment.

 9. Automation of a diagnostic chain process is aimed at providing a consistent quality of material for making diagnoses. Its variation should be analyzed with regard to its influence on the final diagnosis.

 10. The most efficient quality assurance in automated technology is the accuracy afforded by quantification of measurement.

 11. Only VM permits a continuous on-line evaluation of image quality prior to the pathologist’s diagnostic statement [1,20].

The emerging tendency to separate pre-diagnostic workflow steps in the histopathology laboratory from the final stages that are important for pathologists to make diagnoses are likely to create new quality assurance procedures in agreement with the above listed statements. The contribution of VS to logistic and diagnostic inaccuracies is probably less important when compared with tissue preservation and processing, as all molecular biology and genetic investigations depend upon tissue preservation and processing.

### Is Europe ready to implement VS in routine diagnostic surgical pathology?

Many European pathologists can be conservative when working in a strictly regulated financial environment. Financial issues play the main role in all non-research technological or medical investments, and are strictly separated from research and scientific approaches.

In Germany, to my knowledge, several University Institutes of Pathology have already undertaken new efforts to implement VM into routine diagnosis. Financial issues, in particular the high investment and considerable expense of integrating VM into the existing LIS, are hard to overcome due to restrictive administrative business plans of routine medical services. Most of these institutions are already equipped with VS scanners but none of them are used in routine diagnosis.

Discussions on VS image quality, or accuracy of VM based diagnosis, play only a minor role if any at all. All serious reports of telepathology and VS experiences display congruent evidence that VM is at least an equal if not superior diagnostic tool compared to conventional microscopy [1,6,8]. Diagnoses can be evaluated from VS without legal problems to my knowledge.

Experiences of VM applied in routine diagnosis have been reported from one private Institute of Pathology located in Heerlen, The Netherlands [8]. It started with VS based routine diagnosis in 2006; and then an additional University Institute located in Uppsala, Sweden followed. They reported no difficulties in performance or increased diagnostic failures.

Some additional (minor) constraints that might influence the implementation of VM into routine tissue-based diagnosis include:

 1. The new laboratory and LIS configuration have to be approved by a new third party certification, which is again a question of investment.

 2. Glass slides have to be stored in VM as usual, in Germany this is for mandatory for at least 10 years. Herein, VM produces additional costs, and does not save money.

 3. VM implementation only adds a link to the chain of laboratory workflow. It can only be used for detailed workflow monitoring at its end stage, i.e. does not lower the costs of laboratory certification.

 4. VM is an appropriate tool to fasten and improve communication between the laboratory and pathologist. This is only of importance in large institutions.

 5. VM opens the door to communicative medicine. Specific medical information obtained from different sources can be included into VM without substantial extra effort. Again, large institutions will receive the highest benefit of this.

 6. VS scanners are only a tool and are open to human error, in the same sense that a car is. The car does not require specific administrative regulations; wrong driving is the fault of the driver and not of the car.

 7. The scanned VS could be subject to FDA approval or other inspections, although they are viewed continuously by pathologists. The scanner itself is of no harm, and any dysfunction will be immediately noticed, especially in routine diagnostics.

In conclusion, the recognition of the advantages of VM by the appropriate administration requires some intelligence. The idea of the implementation of VM is an unusual consideration, however, if performed, an action which in German would be called “nachhaltig” (its English equivalent roughly translates to “especially good in future taking into account less momentary success”; or to be less precise, “to achieve sustained success”). Most administrations currently seem to be unable to perform or permit a “nachhaltige” action, neither in Germany nor elsewhere in Europe.

However, there remains another option which gives us hope. This is the enormous amount of money that is currently invested by the industry, which hints at future reimbursement by appropriate administrative actions. Fortunately, no proven scientific or other no financial constraints against this action seem to exist as far as I know.

## Competing interest

The author declare that they have no competing interests.
